# Transcriptome-wide profiling and expression analysis of transcription factor families in a liverwort, *Marchantia polymorpha*

**DOI:** 10.1186/1471-2164-14-915

**Published:** 2013-12-23

**Authors:** Niharika Sharma, Prem L Bhalla, Mohan B Singh

**Affiliations:** 1 Plant Molecular Biology and Biotechnology Laboratory, Australian Research Council Centre of Excellence for Integrative Legume Research, Melbourne School of Land and Environment, University of Melbourne, Parkville, Melbourne, Victoria 3010, Australia

**Keywords:** Liverwort, *Marchantia polymorpha* transcriptome, Transcription factor, Evolution

## Abstract

**Background:**

Transcription factors (TFs) are vital elements that regulate transcription and the spatio-temporal expression of genes, thereby ensuring the accurate development and functioning of an organism. The identification of TF-encoding genes in a liverwort, *Marchantia polymorpha*, offers insights into TF organization in the members of the most basal lineages of land plants (embryophytes). Therefore, a comparison of *Marchantia* TF genes with other land plants (monocots, dicots, bryophytes) and algae (chlorophytes, rhodophytes) provides the most comprehensive view of the rates of expansion or contraction of TF genes in plant evolution.

**Results:**

In this study, we report the identification of TF-encoding transcripts in *M. polymorpha* for the first time, as evidenced by deep RNA sequencing data. In total, 3,471 putative TF encoding transcripts, distributed in 80 families, were identified, representing 7.4% of the generated *Marchantia* gametophytic transcriptome dataset. Overall, TF basic functions and distribution across families appear to be conserved when compared to other plant species. However, it is of interest to observe the genesis of novel sequences in 24 TF families and the apparent termination of 2 TF families with the emergence of *Marchantia*. Out of 24 TF families, 6 are known to be associated with plant reproductive development processes. We also examined the expression pattern of these TF-encoding transcripts in six male and female developmental stages in vegetative and reproductive gametophytic tissues of *Marchantia*.

**Conclusions:**

The analysis highlighted the importance of *Marchantia*, a model plant system, in an evolutionary context. The dataset generated here provides a scientific resource for TF gene discovery and other comparative evolutionary studies of land plants.

## Background

Regulation of gene expression is central to all organisms [1] and is imperative for determining the morphology, functional competence, and development of a multicellular organism [2]. This regulation is tightly coordinated by a number of mechanisms, such as DNA methylation [3]; chromatin organization [4]; dimerization; and sequence-specific DNA binding, which is executed primarily by transcription factors (TFs). Depending upon the combinatorial control of protein-protein interactions, a TF may simultaneously function as an activator of one set of genes and a repressor of others [5]. For example, TFs have been known to determine the identity of floral organs in plants [6]. These TFs, referred to as organ identity genes, control the transcriptional regulation of target genes, thereby triggering organ formation in sexual plant reproduction. Via their various actions, these modular proteins play a pivotal role in controlling the spatial and temporal expression patterns of genes in all living organisms.

Usually, TFs are comprised of a DNA-binding domain (DBD) that interacts with the *cis*-regulatory elements of its target genes [7] and a protein-protein interaction domain that facilitates oligomerization between TFs and other regulators [8]. The majority of TFs may be grouped into a number of different families according to their structural features, i.e., the type of DBD that is present within their sequence [5]. Usually, each TF has only one type of DBD, occurring in either single or multiple copies.

Eukaryotes have a more sophisticated transcription regulation mechanism than prokaryotes. Multicellular eukaryotes must address cell differentiation and consequently administer a more enigmatic regulatory mechanism, which uses a large number of TFs [9-12]. Reports have also shown that TF families are strongly conserved across eukaryotic organisms, especially plants [13]. Approximately, 45% of *Arabidopsis* TFs belong to families that are specific to plants [1]. As in animals, TF families have been considerably expanded in plant lineages, suggesting that they are involved in the regulation of clade-specific functions [1,8,14,15]. Thus, plants have more TF genes than animals [13,16]. A significant number of protein-encoding genes are dedicated to regulating the transcription machinery and gene expression [1]. In plants, ~7% of all genes encode for TFs. For example, the genome of *Arabidopsis thaliana* includes 27,416 protein-coding genes (TAIR http://www.arabidopsis.org/), of which 6% (more than 1,700) encode TFs.

The completion of various genome sequencing projects has provided a unique opportunity for comparative studies of transcriptional regulatory networks. Distribution and sequence analyses suggested that TF genes in plants evolved via genome duplication [17], exon capture, translocation, and mutation. The retention of duplicated TF genes led to gene family expansions, which further complicated the genomes of higher plants [18]. TF families that have significantly expanded in the past 600–100 million years are mainly the MADS box proteins, basic-region leucine-zipper proteins (bZIP), and the MYB and bHLH families [8,19,20].

Plants and animals are known to have originated from a common ancestor. Structural conservation of TF DBDs among plants and animals suggests that these domains may have originated before these two eukaryotic kingdoms diverged. Little structural conservation has also been reported among different eukaryotic TFs. This suggests that eukaryotes use only a limited number of DBDs to achieve various regulatory purposes, in combination with other functional activation domains. Thus, TFs may be viewed as molecular switches that link signal transduction pathways to gene expression [7]. The function of a few TF families has remained conserved between plants and animals separated by over a billion years of evolution [1]; one example is the E2F family, which controls basic cell cycle functions [1,21]. On the other hand, many TF families may exhibit altered or diverse functions due to minor sequence changes in different plant and animal lineages [11]. Thus, these evolutionary changes in sequences and TF functions may complicate the detection of paralogous/orthologous relationships between organisms.

Liverworts are among the earliest diverging plant lineages, thus constituting a sister group to all other land plants [22-27]. The bryophyte fossil record shows that liverworts are at least 475 million years old [28]. *M. polymorpha* is a common liverwort with a wide distribution around the world and is one of the most intensively studied bryophytes. Because they belong to the clade of the most basal plant lineages, liverworts occupy a very important position with respect to understanding early land plant evolution [29]. No evolutionary study can be complete without data from *Marchantia*. Unfortunately, only minimal genomic information has been available for this bryophyte, until now. Although some expressed sequence tags (ESTs) have been produced and some male and female gene-based markers have been developed, full-fledged functional genomics studies in liverworts have not been initiated. Since *M. polymorpha* is a dioecious plant, ESTs have been generated [30,31] in an attempt to identify key genes involved in sex differentiation mechanisms and the development of male and female plants but are limited in coverage. Here, we present the entire repertoire of regulatory factors in this liverwort for the first time and predict a set of TF-encoding transcripts in *M. polymorpha* on the basis of stringent sequence similarity with known TF genes. Sequence comparisons alone would not have provided the appropriate information regarding the alterations of TF function during evolution; hence, we also examined the expression profiles of the TF-encoding transcripts in *M. polymorpha*. In this study, we also focused on the evolution of TF gene families based on a comprehensive comparison of TF gene distribution in liverworts, mosses, higher plants, and their algal ancestors.

## Results and discussion

### Identification of TF-encoding transcripts through transcriptome sequencing and *De novo* assembly

The transcriptome of *M. polymorpha* was sequenced from RNA isolated from six different male and female tissues, as described in materials and methods section and shown in Additional file 1, using short reads on an Illumina HiSeq™ 2000 platform (Sharma et al., unpublished observations). The chosen tissue samples for RNA isolation and sequencing represented the most comprehensive repository of vegetative and reproductive stages of both male and female gametophytic tissues. The *Marchantia* transcriptome dataset generated from this study is a new source for the identification of novel regulatory transcripts and has provided a glance of their expression profiles in vegetative and reproductive tissues.

Approximately 80 million paired-end sequence reads, each 90 bp in length, were generated from RNA sequencing (Sharma et al., unpublished observations). Low-quality reads were filtered out before assembly. *De novo* transcriptome assembly was performed with Velvet [32] and Oases [33] using the same parameters used by Garg and colleagues for their transcriptome assembly [34]. *De novo* assembly of the *Marchantia* transcriptome resulted in a total of 46,533 non-redundant (NR) transcripts from 46,070 predicted loci. The sequence dataset generated is deposited at NCBI in the Short Read Archive (SRA) database under accession number SRP029610.

The total genome size of *M. polymorpha* was estimated to be 280 Mb based on flow cytometry, and the total number of genes was estimated to be ~20,000 [35]. In this study, 46,533 transcripts from 46,070 loci, potentially representing an estimated number of genes, were predicted from the transcriptome data of *M. polymorpha*. This number likely includes the alternatively spliced variants and non-coding transcripts. In fact, only 20,000 out of 46,533 transcripts generated BLASTX hits, with an E-value cut-off of 1e-05, against the protein sequences of embryophytes that were extracted from the NR NCBI database (Sharma et al., unpublished observations). Hence, we assume that most of the *Marchantia* genes, including TF genes, were detected by our RNA-Seq data. Our results indicate that the obtained transcript dataset may be fragmentary. Thus, the number of transcripts/genes encoding for TFs is likely to be fewer than what is presented in the data below. Further, the genome sequence information for *Marchantia* may provide more information about the fragmentation of transcripts in this liverwort.

**Figure 1:**
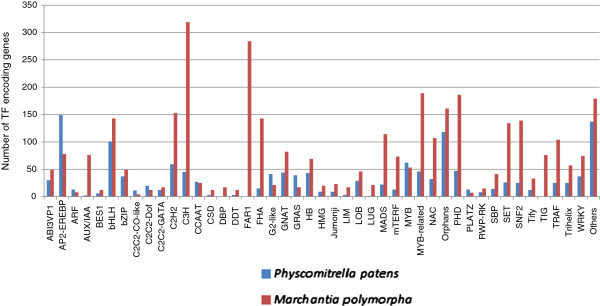
**Distribution of *Marchantia *transcripts in different transcription factor families.** A bar graph indicating the number of TF-encoding genes in *Marchantia polymorpha* and *Physcomitrella patens* distributed in various TF families. If the number of genes/transcripts encoding for a particular TF family is less than 12, those families are listed in others category.

The assembled NR transcripts of *Marchantia* were compared with known TF gene sequences of other sequenced plants listed in PlnTFDB [36] using BLASTX. In total, 3,471 putative *Marchantia* TF-encoding transcripts, distributed in at least 80 families, were identified, representing 7.4% of the total *Marchantia* transcripts detected in our study. Major TF gene families are depicted in Figure 1. The organization of TF families in *Marchantia* resembled that of *Physcomitrella patens*[36-39].

Hence, the description of TF-encoding transcripts from *Marchantia* provided insight into the organization and biological functions of TFs in lower plants as well as their evolution. From a biotechnological standpoint, TF identification is useful for studying the transcriptional regulatory switches involved in plant development and reproduction and in generating responses and sequential adaptations to the changing environment.

### Comparison of TF-encoding genes in plants and their algal ancestors

In the present study, we first summarized the knowledge of TF-encoding genes in plants and algae, while updating the classification of *Marchantia* TF-encoding transcripts and their categorization in all 80 different TF families. PlnTFDB [40] includes 85 families of TFs and TRs from 20 sequenced plant species other than liverworts, ranging from unicellular red and green algae to highly complex angiosperms, thereby including >1.6 billion years of gene regulatory network evolution and encompassing 26,184 distinct proteins. Sequence data showing the number of TF encoding genes in red algae, green algae, *Selaginella*, *Physcomitrella, Chlamydomonas*, and other higher plants is listed in Table 1.

**Table 1:** Number of genes/transcripts encoding TFs for various organisms

**Plant type**	**Class**	**Organism**	TF encoding gene/*transcripts
Red algae	Rhodophytes	*Cyanidioschyzon merolae*	157
		*Galdieria sulphuraria*	213
Green algae	Prasinophytes	*Micromonas pusilla CCMP1545*	309
		*Micromonas sp. RCC299*	350
		*Ostreococcus lucimarinus*	255
		*Ostreococcus tauri*	227
	Chlorophytes	*Chlorella sp. NC64A*	321
		*Chlamydomonas reinhardtii*	367
		*Coccomyxa sp. C-169*	283
Liverwort	Marchantiopsida	*Marchantia polymorpha*	3471*
Moss	Bryopsida	*Physcomitrella patens*	1423
Spike-moss	Lycopodiophyte	*Selaginella moellendorffii*	949
Angiosperms	Monocots	*Oryza sativa subsp. indica*	2502
		*Oryza sativa subsp. japonica*	3119
		*Sorghum bicolor*	2312
		*Zea mays*	5246
	Eudicots	*Arabidopsis lyrata*	2268
		*Arabidopsis thaliana*	2757
		*Carica papaya*	1538
		*Populus trichocarpa*	3078
		*Vitis vinifera*	1799

Numbers of TF encoding genes were taken from PlnTFDB for all organisms except *M. polymorpha*. *For *M. polymorpha*, it is number of TF encoding transcripts generated from our transcriptome sequencing dataset.

Data presented in Table 1 show that the number of genes encoding TFs is the smallest for algae; the number increases from liverworts to mosses, and increases further in monocots and dicots. More complex organisms execute complex mechanisms to control gene expression by employing a greater number of TFs [2,9-12,15,41]. In eukaryotes, an appreciable number of protein-coding genes encode TFs. The number of TF-encoding genes ranges from 2–9% of the total protein-coding genes of the 20 organisms considered. As expected, based on published reports, the smallest number of TF genes was found in the most primeval organisms e.g., *Chlamydomonas* and *Physcomitrella*, where TF genes were found to be 2%–4% of the total genes annotated. In higher plants, the greater complexity of form and function presumably mandates an increased number of TF genes (e.g., monocot and dicot plants have 5–9% TF genes) [13]. This was clearly demonstrated in some earlier reports, which are summarized in Table 2. The number of total predicted protein-coding genes and the number of predicted TF genes identified are also indicated.

**Table 2:** TF gene percentages for various algae, liverworts, mosses and higher plants

**Plant type**	**Organism**	**Predicted total gene number**	**Predicted TF gene number**	**Predicted TF gene percentage**	**References**
**Red algae**	*Cyanidioschyzon merolae*	5,331	157	2.9	[40,42]
	*Galdieria sulphuraria*	5,872	213	3.6	[40,43]
**Green algae**	*Micromonas pusilla CCMP1545*	8,616	309	3.5	[40,44]
	*Micromonas sp. RCC299*	10,109	350	3.4	[40,45]
	*Ostreococcus lucimarinus*	7,651	255	3.3	[40,46]
	*Ostreococcus tauri*	7,725	227	2.9	[40,47]
	*Chlorella sp. NC64A*	9,791	321	3.2	[40,48]
	*Chlamydomonas reinhardtii*	16,709	367	2.1	[13,40]
	*Coccomyxa sp. C-169*	9,851	283	2.9	[40,49]
**Liverwort**	*Marchantia polymorpha*	~20000 (46,533 transcripts)*	3,471*	7.4	
**Moss**	*Physcomitrella patens*	35,938	1,423	3.9	[39,40]
**Spike-moss**	*Selaginella moellendorffii*	22,285	949	4.2	[40,50]
**Angiosperms**	*Oryza sativa subsp. indica*	55,615	2,502	4.4	[40,51]
	*Oryza sativa subsp. japonica*	50,000	3,119	6.2	[40,52]
	*Sorghum bicolor*	36,338	2,312	6.3	[13,40]
	*Zea mays*	125,435	5,246	4.1	[13,37]
	*Arabidopsis lyrata*	32,670	2,268	6.9	[40,53]
	*Arabidopsis thaliana*	32,825	2,757	8.3	[13,40]
	*Carica papaya*	27,793	1,538	5.5	[40]http://www.plantgdb.org/CpGDB/
	*Populus trichocarpa*	45,555	3,078	6.7	[13,40]
	*Vitis vinifera*	30,434	1,799	5.9	[13,40]

List of number of TF-encoding genes and total number protein-coding genes in various organisms. *denotes the number of transcripts encoding for TFs for *M. polymorpha*. This number also includes the alternatively spliced variants and non-coding transcripts whereas else all refers to the number of protein-coding genes.

Existing knowledge of plant TF genes was acquired from various studies conducted on an exemplar genetic model in plant biology—*Arabidopsis thaliana*. Despite *Arabidopsis* being an important and very useful plant model for studying various developmental processes and regulatory mechanisms common to all higher plants [13], it lacks certain traits that are concomitant with the evolutionary movement of plants from aquatic conditions to land, such as the loss of genes associated with an aquatic environment and acquisition of genes for tolerating terrestrial stresses. These traits are of immense value to lower plants, and this may support the concept of evolution of plants from their algal ancestors. Hence, it was of great interest to perform a more comprehensive comparative analysis of TF genes between alga, moss, spike moss, liverwort, and higher plants. We considered the identity of organisms when evaluating gene family sizes, as various organisms are reported to have different rates of gene duplication and retention, and differences in gene content may reflect species-specific adaptations [39].

**Figure 2:**
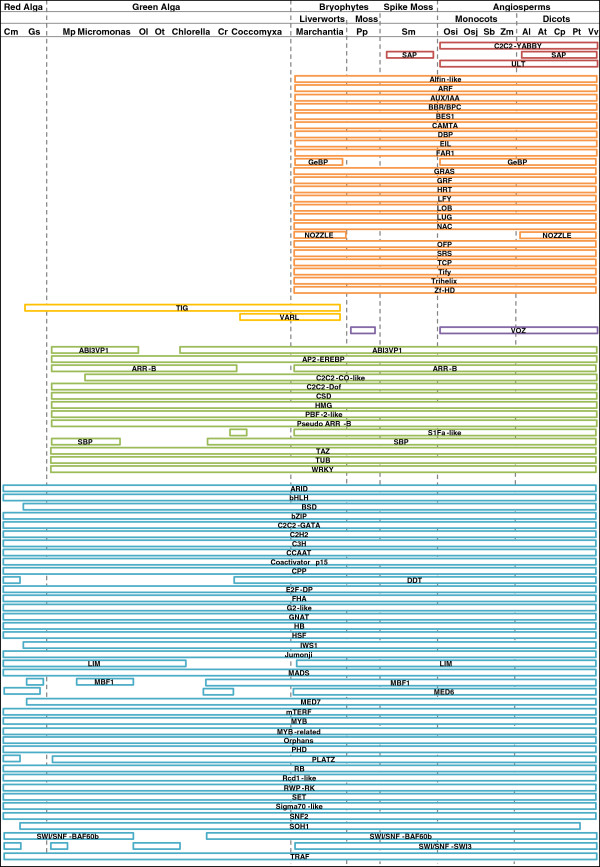
**A comparative analysis of distribution of TF families in different groups of organisms like algae, liverworts, mosses and higher plants (monocots and dicots).** Presence or absence of various TF families in different groups of organisms: red alga, green alga, liverwort, moss, spike-moss and angiosperms (monocots and dicots). Different colour horizontal bars used to indicate different groups of TF families arising from different groups of organisms. List of organisms included in the study are: Cm: *Cyanidioschyzon merolae*; Gs: *Galdieria sulphuraria*; Mp: *Micromonas pusilla*; Micromonas: *Micromonas sp. RCC299*; Ol: *Ostreococcus lucimarinus*; Ot: *Ostreococcus tauri*; Chlorella: *Chlorella sp. NC64A*; Cr: *Chlamydomonas reinhardtii*; Coccomyxa: *Coccomyxa sp. C-169*; *Marchantia*: *Marchantia polymorpha*; Pp: *Physcomitrella patens*; Sm: *Selaginella moellendorffii*; Osi: *Oryza sativa indica*; Osj: *Oryza sativa japonica*; Sb: *Sorghum bicolor*; Zm: *Zea mays*; Al: *Arabidopsis lyrata*; At: *Arabidopsis thaliana*; Cp: *Carica papaya*; Pt: *Poplus trichocarpa*; Vv: *Vitis vinifera*.

Figure 2 shows 85 TF families, color-coded according to the lineage of land plants in which they were commonly found. A strikingly important observation made from the analyzed comparative dataset is that, out of the 85 gene families taken into consideration, 24 appear to originate as liverworts evolved (marked as orange blocks). These families are present in all land plants, including liverworts, but are absent in red algae (rhodophytes) and green algae (chlorophytes): Alfin-like, ARF, AUX/IAA, BBR/BPC, BES1, CAMTA, DBP, EIL, FAR1, GeBP, GRAS, GRF, HRT, LFY, LOB, LUG, NAC, NOZZLE, OFP, SRS, TCP, Tify, Trihelix and zf-HD. An initial report stated that these 21 TF families arose within the earliest land plants or in their aquatic ancestor [2]. However, taking *Marchantia* into consideration, given that it is the earliest diverging lineage, transcriptome sequencing provided us with new findings. The numbers of TF-encoding genes in all studied organisms are given in Additional file 2. Some TFs, which originated together with the evolution of liverworts, contribute to the stress tolerance capacity of plants: for example, CAMTA [54] and Alfin-like [55] regulate salt tolerance; ARF [56] and AUX/IAA [57] play roles in auxin regulation; EIL [58] is known for ethylene signaling in higher plants; and GRF [59], LFY [60], LOB [61], LUG [62], NAC [63], NOZZLE [64], OFP [65], and Tify [66] regulate meristem elongation, flowering initiation, and flowering organ development [6]. Trihelix TFs are known to be involved in diverse functions in seed plants, such as abiotic stress tolerance [67], ploidy-dependent cell growth [68], repression of seed maturation [69], and perianth architecture [70]. Also, the lack of these TF families in the algal genomes studied here indicates their possible involvement in transcriptional regulation of cell-to-cell interactions in the multicellular liverwort body plan.

Out of the 24 families reported above, six are known to play roles in sexual plant reproduction [60,62,64,66,71,72], suggesting that these TFs are likely crucial in accounting for the increasing complexity of reproductive processes in plants. In this study, we report that 24 TF families are likely to be associated with the divergence of land plants from their aquatic ancestors and the evolution of complex reproduction processes in plants. The addition of *Marchantia* in the study of TF gene evolution clarify these basal plant TFs and thus make a valuable contribution in this field.

Embryophytes, which began to branch off approximately 450 million years ago, carry a suite of genes governing their adaptation to a terrestrial environment. Bryophytes, consisting of hornworts, mosses, and liverworts, are the extant representatives of early diverging lineages, and liverworts hold a prominent phylogenetic position. Liverworts marked the transformation from an aquatic to a terrestrial environment, which involved variation in water availability and temperature, as well as increased exposure to radiation. Consequently, adaptations necessitated striking changes in their body plan [29,73] and modifications to cellular, physiological, and regulatory processes. Primary adaptations included enhanced osmoregulation and osmoprotection, desiccation and freezing tolerance, heat resistance, the synthesis and accumulation of protective ‘sunscreens’, and enhanced DNA repair mechanisms. Fossil evidence also suggested that early land plants were structurally similar to extant bryophytes [74], likely had a dominant haploid phase, and were dependent on water for sexual reproduction because they had mobile male gametes. Hence, *Marchantia* retained the characteristics of extant bryophytes and, at the same time, acquired many new TFs to administer a higher degree of regulation and complexity. Hence, the comparison revealed predicted genomic changes that were concomitant with the evolutionary movement to land, including a general increase in gene family complexity, a loss of genes associated with aquatic environments, the acquisition of genes for tolerating terrestrial stresses, and the development of the auxin and abscisic acid signaling pathways for coordinating multicellular growth and dehydration.

In contrast to the above observation, the appearance of *Marchantia* also marked the cessation of two TF families, TIG and VARL, depicted as yellow blocks in Figure 2. TIG [75] is a family that is present in liverworts, red and green algae, and *Chlamydomonas*, but is totally absent in higher plants. VARL similarly is found in *Marchantia* and *Chlamydomonas*, reported to be present in algal ancestors, but completely lost in later lineages of land plants [76]. While representing only two TF families, this observation suggests that the shared ancestral TF encoding genes between algae and liverworts were lost along higher plant lineages like flagellar associated proteins (FAPs) [77]. Unfortunately, the functions of only a few TF genes in lower plants are known. Interestingly, *Marchantia polymorpha* shows the presence of a single homologue of these MADS box genes. MIKC* MADS-box proteins are essential for male gametophyte development in *Arabidopsis.* Interestingly, *MpMADS1* for instance was found to form a homodimeric DNA-binding complex, which is in contrast to the *Arabidopsis* proteins that are functional only as heterodimeric complexes. MADS box genes are also present in charophycean algae, the closest relative of land plants. In charophycean algae, these genes functioned in haploid reproductive cell differentiation [78,79]. Charophytes evolved many ancestral traits (cellulose synthase, plasmodesmata, apical growth, placenta) that proved important for survival in a terrestrial environment but never left the aqueous environment. Thus, it will be great interest to compare the transcriptional repertoire of *Marchantia* with that of charophycean green algae; however available algal transcriptomes are reported to be incomplete, and currently available results are only based upon preliminary studies [73]. KNOX/BELL is a TF family reported to be present in the green algal lineage [80], whereas C3HDZ [81], C4HDZ, and the WOX-type homeodomain proteins [77] are present only in some charophycean algae. Some components of the auxin transcriptional response and ethylene signaling pathways have also been suggested to originate within charophycean algal lineage [73]. Therefore, the considerable effort needed to define the role of TF genes in lower plants like liverworts and charophycean algae may be well invested. The new approaches of high-throughput RNA-Seq and genome-based analysis could easily extend investigations of these groups to include a thorough identification and classification of currently uncharacterized but crucial TF gene populations [82]. Highlighting similarities and differences in TF gene populations among lower and higher plants are expected to help us to better understand the evolution of regulatory elements.

TF families marked with olive blocks in Figure 2 are absent in red algae but present in green algae through to angiosperms: ABI3VP1, AP2-EREBP, ARR-B, C2C2-CO-like, C2C2-Dof, CSD, HMG, PBF-2-like, Pseudo ARR-B, SBP, TAZ, TUB, and WRKY. We also observed that TF families depicted as red blocks—C2C2-YABBY, SAP and ULT—are found only in angiosperms [2]. YABBY determines the abaxial or adaxial cell fate, which appears to be characteristic of higher plants [83]. SAP is a sterile apetala TF that is essential for flower development [84]. ULT is also known to promote shoot and floral meristem development in plants [85]. Data also suggested that the VOZ family, which plays a role in pollen development, evolved with *Physcomitrella*[86]. Such a study of TF- and TR-encoding transcripts is crucial to determine the lineage- or species-specific TFs that control specific developmental processes, as well as for studying evolutionary processes, such as speciation and adaptation. The functions of all TFs are listed in Additional file 3. The functions of TF families that arose with *M. polymorpha* are summarized in Additional file 4.

Furthermore, we observed that *Marchantia* TFs and TRs corresponded to a few functionally conserved categories common among other plants. The conservation of TF gene families signified the presence of similar gene regulatory machinery in liverworts and higher plants. However, there was evidence of evolutionary expansion and contraction events of for some TFs, which contributed to the presence of some lower plant-specific and some higher plant-specific traits in liverworts. Since TF gene family sizes differ among different organisms, primarily because of variable rates of gene duplication and retention, these gene content disparities might indicate species-specific adaptations [39]. On the basis of comparing the number of genes coding for a particular TF in the 20 organisms studied here, expansion events have been delineated in the ABI3VP1, AP2-EREBP, bHLH, C2H2, C3H, DDT, HB, MADS, MYB, MYB-related, Orphans, PHD, PLATZ, SET, SNF2, SWI/SNF-BAF60b, TCP, Tify, TIG, TRAF, and WRKY families. The expansion of TF gene families encoding regulatory proteins was naturally correlated with increased convolution in multicellular organisms [1]. For example, the DDT TR family is not well characterized; is known to contain a DNA-binding domain [87]; is present in single copy in unicellular organisms and in the land plants encodes for more than one DDT encoding gene. These features suggest their involvement in the regulation of transcription in multicellular species. A putative homolog of HB family has also been shown to be involved in differentiation of preprocambial cells into xylem during leaf vein formation [88] in *Arabidopsis*, but vascular cambium is absent in bryophytes. We therefore suggest that this TF has a role in the development and evolution of the multicellular body plan.

Contraction was shown in the Alfin-like, BBR/BPC, BES1, BSD, CAMTA, Coactivator p15, CPP, CSD, DBP, E2F-DP, EIL, FAR1, FHA, SWI/SNF-SWI3, and TAZ families. These results are delineated in Additional file 2. Considering the distribution of genes among the 85 TF families identified, only one family was found to be specific to algae, and three families were found to be specific to higher plants. Seventeen families indicated the likely conservation of TF families among land plants.

### bHLH and MYB families as an example of TFs evolution

The expansion of gene families in plants is most frequently linked to genome duplication events. Genome duplications are reported to confer a competitive advantage under changing environmental conditions and to enhance the diversification potential of a lineage [41]. The MYB and bHLH families present examples of how gene duplication and divergence in a particular group of TFs correlate with the morphological and metabolic diversity that distinguish the higher plants [1,89], which expanded dramatically in higher plants. There are approximately 166 MYB and 177 bHLH genes in *Arabidopsis*, and approximately 127 MYBs and 195 bHLH genes, respectively, in rice. In contrast, *Chlamydomonas* has only 10 MYB genes and 8 bHLH genes. Such TF family expansions have been reported to be greater in plants than in animals [1,8,20]. Gene duplication events are purported to lead to three successful functional outcomes: i) one of the duplicated genes can preserve the same function as before duplication (sub-functionalization), ii) one of the duplicated genes can acquire a new function (neo-functionalization), or iii) one of the duplicates can become non-functional [1,90]. A recent report stated that a total of 3,814 TF-encoding genes were present in all plant lineages and defined the minimum set of genes that were likely to be present in the common ancestor of all green plants and their descendants, including genes that were essential for plant function [50]. The transition from single-celled green algae to multicellular land plants approximately doubled the number of genes, with the acquisition of 3,006 new genes. The transition from non-vascular to vascular plants was associated with a gain of far fewer new genes (516) than the transition from basal vascular plants to basal euphyllophytes, which include angiosperm descendants (1350). These numbers show that the evolution of traits specific to angiosperms required the evolution of about three times more new genes than the transition from a plant having a dominant gametophyte, leafless, and non-vascularized sporophyte to a plant with a dominant, vascularized and branched sporophyte with leaves [50].

bHLH proteins are an ancient family detected in fungi, plants and animals but not in prokaryotes. It is also reported that expansion in the bHLH family occurred after the split between green algae and land plants, but before the origin of mosses. After the divergence of mosses from vascular plants, a second expansion took place that likely correlated with the specialization of vascular and flowering plants [1]. Our analysis of various land plants, chlorophytes, red algae and *Marchantia* confirmed this hypothesis in liverworts and suggested that ancient plants had one or only a few bHLH genes and that all modern plants, including *Marchantia,* have bHLH proteins that are descended and radiated from these predecessors by a process that involved a substantial number of gene duplication events [91,92]. Most of the bHLH proteins identified have been functionally characterized in *Arabidopsis* with evidence of remarkable functional expansion, including roles in the regulation of fruit dehiscence, carpel, anther and epidermal cell development; phytochrome signaling, flavonoid biosynthesis, hormone signaling and stress responses [1]. bHLH proteins act as transcriptional activators or repressors and have either very broad or very restricted expression patterns, presumably reflecting in the latter case key roles in cell identity and specialization.

The presence of MYB repeats has been identified in all eukaryotic organisms studied to date divided into a number of key subfamilies [89]. R2R3-MYBs form the largest group of plant MYB factors, and hundreds of its members containing the conserved domain have been investigated in all the terrestrial plants [1]. This primordial family of TFs is believed to have existed before the divergence of plants and animals [93]. Rabinowicz and colleagues reported that plant MYB proteins underwent an extensive expansion around the time of the origin of land plants, before the separation of monocots and dicots [94]. Our study also identified the presence and expansion of the MYB gene family in liverworts and subsequently in higher plants (monocots and dicots) (Additional file 2). Hence, the expansion probably occurred in response to selection for the regulation of processes related to increasing tolerance to freezing, drought and salt stress in sessile land plants [1].

Transcription factors of the same family may have distinct actions because of differences in their regulatory domains [95]. However, TF genes of the same family and from different organisms may exhibit structural and functional similarities, thereby conveying the messages that they evolved from a common ancestor. Gene duplication events that occurred in various organisms undoubtedly played a vital role during this evolution. After duplication, TF gene organization may have been modified by translocation, causing related family members to be scattered throughout the genome or aggregated on one chromosome [95]. Morphogenesis, development and habitat behavior of different plant species rely on different patterns of gene expression. Hence, the TF gene repository of a given plant species, their expression profiles and their function reflect the unique characteristics of the species [15].

### Expression patterns of TF encoding transcripts of *M. Polymorpha*

**Figure 3:**
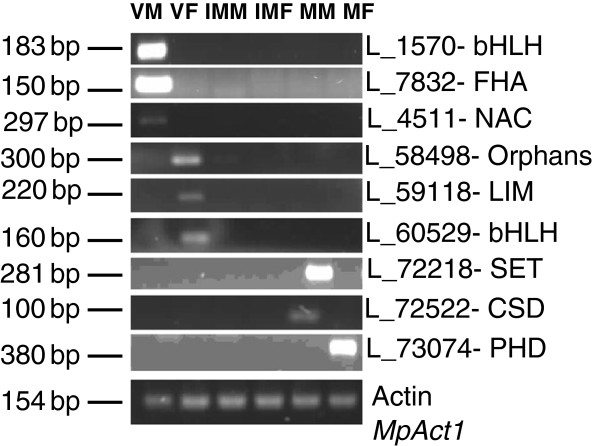
**RT-PCR analysis.** Gel figures demonstrating confirmatory RT-PCR analysis for transcripts specifically expressed in one of the six stages in our transcriptome data encoding for various Transcription Factors. The experiments were performed on total RNA extracted from (1) vegetative thallus male (VM), (2) vegetative thallus female (VF), (3) immature male reproductive (IMM), (4) immature female reproductive (IMF), (5) mature male reproductive (MM) and (6) mature female reproductive (MF) tissues. Actin is used as a positive control expressed in all 6 stages.

Based on the RPKM method, we obtained expression values for all *Marchantia* TF-encoding transcripts (Additional file 5). The dataset contained a wide range of expression levels for *Marchantia* TF transcripts, depending upon tissue type or particular developmental time. In the dataset, 96 out of 3,471 transcripts were expressed in only one stage (Additional file 5). These specifically expressed TFs were interesting, because they may be involved in defining the precise nature of individual tissues. Some candidates of specifically expressed transcripts were validated by semi-quantitative RT-PCR and are shown in Figure 3. Variable expression patterns were observed, including one stage-specific, vegetative stage-specific, reproductive stage-specific, and constitutive expression.

### TFs specifically expressed in vegetative stages

56 TF encoding transcripts exhibited preferential expression in vegetative tissues of both male and female plants of *Marchantia*. Of these TF encoding transcripts, 42 showed vegetative thallus male (VM) specific expression whereas only 3 had vegetative thallus female (VF) specific expression and 11 were found in both sexes of thalli. The top highly expressed VM candidates encode for bHLH, FHA and NAC families with RPKM values of above 100 whereas VF expressing TF genes have comparatively low RPKM values with the highest having a RPKM value of 24 and these encode for Orphans TF family. A list of transcripts expressed in vegetative tissues and their respective TF families is provided in Additional file 6.

### TFs specifically expressed in reproductive stages

138 transcripts in total were found to have preferential expression in all four reproductive tissues. 11 and 37 transcripts depicted specific expression in the mature reproductive stages mature reproductive male (MM) and mature reproductive female (MF) respectively, and only 1 and 2 transcripts, displaying immature reproductive male (IMM) and immature reproductive female (IMF) specific expression. Overall there were 189 transcripts that showed preferential expression in any of the four reproductive stages of *Marchantia*. The most frequently represented reproductive-associated TFs belonged to the bHLH, C3H, MYB, PHD, SET, TIG and WRKY families of TFs. A complete list of transcripts expressed in reproductive tissues and their respective TF families is given in Additional file 6.

### Correlation in expression patterns of transcripts in different TF families

Correlations between the expression patterns of TF-encoding genes (given in Additional file 5) within each TF family were investigated using Statistical Analysis Software (SAS) version 9.2. Significant positive/negative correlations (having *p*-value < = 0.05) of expression patterns were observed between TF-encoding genes in 63 TF families as reported in Additional file 7. For example, a) six transcripts are seen correlatively specifically expressed in vegetative stages of *Marchantia* and encode for the FAR1 TF family. FAR1 TF is known to modulate phyA-signalling in plants. As these photoreceptors are sensitive to light in red and far-red regions of the visible spectrum [96], *Marchantia,* like *Arabidopsis,* seems to use photoreceptors to regulate the time of sexual reproduction based on day length. *Marchantia* has been reported to need far-red light to initiate the growth of sexual reproductive structures [97,98]. b) Eight transcripts coding for MYB-related TFs are specifically expressed in vegetative tissues. Since MYB-related TFs comprise a small family with a central role in controlling cellular proliferation and commitment to development [99], they seem involved in similar functions in this liverwort also. c) Some transcripts depicted vegetative specific expression and encode for Orphans TF promoting initiation/transition to flowering [100]. As the gene coding for Orphans in *Arabidopsis* has been found to be expressed in collective leaf structure, flowers, plant embryos, seed and shoots, playing a role in floral meristem determinacy and flower development [101], we suggest these transcripts are involved in reproductive transition determinacy in *Marchantia.* d) 11 transcripts coding for bHLH TF are found to be specifically expressed in MF reproductive stage in *Marchantia*. bHLH proteins are found both in plants and animals and are known to be involved in the regulation of a wide variety of essential growth and developmental processes [91]. bHLH TFs also appear to be involved in carpel development and fruit dehiscence [102]. This supports the involvement of bHLH proteins in the formation of archegoniophores and developing sporophytes in *Marchantia*. e) Eight transcripts showing specific expression in reproductive stages coding for WRKY family are also found. WRKY proteins are known to play significant roles in responses to biotic and abiotic stresses, senescence and pathogen defense [103]. In *Marchantia*, WRKY proteins are transcriptional regulators that are proposed to play a role in proper cellular responses to internal and external stimuli. Other transcripts showing preferential expression pattern for reproductive stages code for AP2-EREBP – a regulator of floral organ identity [104], HB which is involved in cell differentiation and controls cell-growth [105], LOB which functions in plant development in lateral organs like the leaf or flower [61], MYB which controls cellular proliferation and the commitment to development [99], PHD which controls chromatin or transcription [106], SET which is involved in histone methylation [107], and TIG which is involved in DNA binding [75]. Thus, these TFs are proposed to play similar roles in *Marchantia.*

In plants, the manifestation of fundamental biological processes and proper development requires some genes to be expressed constitutively, while others are expressed in a specific spatio-temporal pattern (organ-limited, stimulus-responsive, development-dependent, and cell-cycle specific manners). Both patterns of expression rely on the interaction of TFs with *cis*-acting elements or with other TFs for the regulation of cell activities. Hence, any change in the expression profile of TF genes in tissues normally leads to dramatic changes in plant development, and structural changes to these genes may signify an important evolutionary force [95]. As a practical approach, studying the expression pattern of these TF-encoding transcripts in liverworts provides us with strong evolutionary support for models and emphasizes the importance of this model plant system.

### Putative functions of TF-encoding transcripts

3,471 TF-encoding transcripts were subjected to a BLASTX search against the non-redundant (NR) database of the NCBI (National Center for Biotechnology Information). The BLASTX search used an E-value cut-off of 1e-05. Out of 3,471 transcripts, 3,395 (97.8%) resulted in hits, supporting that these are the protein-coding genes. 94.8% of 3,395 transcripts resulted in hits with plants. A list of BLASTX hits is provided in Additional file 8.

### qPCR validation

**Figure 4:**
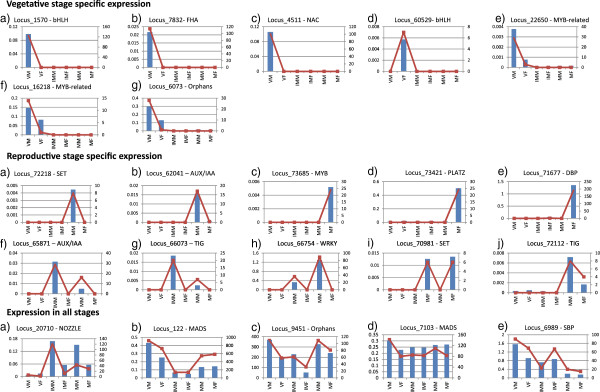
**Real-time RT-PCR expression profiles of selected transcripts coding for transcription factors.** VM – Vegetative thallus Male, VF – Vegetative thallus Female, IMM – Immature reproductive Male, IMF – Immature reproductive Female, MM – Mature reproductive Male and MF – Mature reproductive Female tissues. All reproductive stage tissues referred to antheridiophores and archegoniophores as described in materials and methods. Y-axis on the left side of graphs shows scale for qPCR values and on the right side shows scale for RPKM values.

qPCR analysis was used to compare the expression of selected variably expressing transcripts across a spectrum of tissues, including vegetative, immature, and mature reproductive stages. Transcripts displaying consistent expression across the spectrum of cells were taken as reference genes. Homologues of actin (*MpACT1*) and CDPK (*MpCDPK*) exhibited variable expression in six considered stages when checked by qPCR, as shown in Additional file 9. Hence, CDPK and actin were not taken as reference genes. Instead, based on the RPKM values, a transcript having consistent expression was selected as the reference gene and was cross-checked by qPCR as well (Additional file 9). qPCR results confirmed the *in-silico* calculations for the RPKM values of the dataset for most of the transcripts, as shown in Figure 4. The *de novo* assembled *Marchantia* TF expression data presented here will also be beneficial for performing other functional genomics and comparative genomic studies.

Our *in silico* inspection of the expression patterns of these TF-encoding genes in different vegetative and reproductive tissues suggested tissue-specific and/or stress-responsive attributes in accordance with their expression patterns. The tissue-specific expression profile of a gene could also be used to discuss the combinatorial usage of TFs for dictating the transcriptional program of different tissues. Members of different TF gene families appear to differ in their time and level of expression as they responded to multiple environmental signals and different developmental signs. Consequently, specific lower-plant traits may derive from some unique TF gene expression patterns. Additionally, it is possible that the same TF gene family members variably express in different plants [95]. Hence, the differential expression of similar TF genes upon exposure to contrasting environmental stimuli could be due to *cis*-acting elements. Clearly, the regulation of TF gene expression and function involves a vital network of interrelated processes.

### Statistical analysis

**Figure 5:**
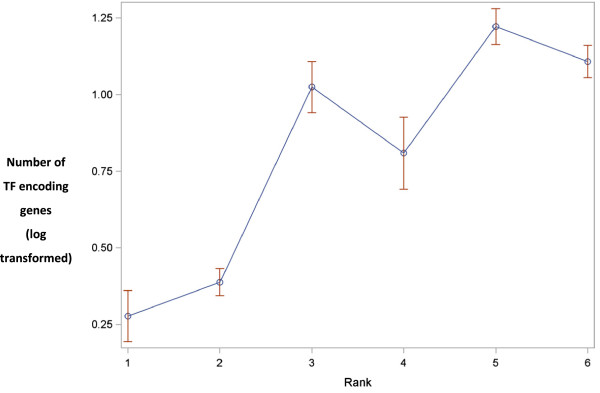
**Statistical analysis.** Ranks 1 & 2 represents red and green alga respectively, 3 & 4 represents *Marchantia* and Moss, Spike moss, *Physcomitrella* respectively and 5 & 6 represents monocots and dicots respectively.

Analysis of variance showed highly significant differences among ranks (*p* <0.0001), in terms of the number of genes coding for TFs, as depicted in Additional file 10. The number of TF-encoding genes appear to increase significantly with organism rank, and thus complexity of the organisms involved. The comparisons of ranks using Gabriel’s comparison limits revealed three major groups. The two most primitive organisms (ranks 1 and 2) had a similarly few number of TF-encoding genes. Organisms classified as rank 5 and 6 (most developed) exhibited a similarly high number of TF-encoding genes. Organisms in rank 3 and 4 showed medium numbers of genes and were placed in between these two extremes, as shown in Figure 5. The results of variance showed that nearly 59% of the total variation in the number of genes coding for TFs was between organisms. Differences between ranks contributed to 39% of the variation, and only 2% variation existed between organisms grouped within a given rank.

## Conclusions

Liverworts as the sister of all land plants represent the basal lineage of land plants, providing a unique perspective on the regulatory origin of TFs and the genetic complexity of terrestrial plants. *Marchantia*, among the liverworts, is particularly easy to grow, transformable, and may prove to be a crucial model for future study of the origin of regulatory genetic systems. The availability of the complete genomic sequences of an increasing diversity of important plant species has provided us with a unique opportunity for comparative studies on the expansion and contraction of TF families. The expansion of regulatory protein numbers and interactions, as well as changes to their spatial and temporal expression, constitute part of the evolutionary process that has led to increasingly complex organisms.

The comparison of *Marchantia* TF genes to other sequenced plant genomes reveals the emergence of new TF families within *Marchantia* that have been preferentially retained and have particularly diversified in higher plants. Among these, such TF families as GRAS, LFY, LUG, NOZZLE, Tify and Trihelix play important roles in sexual plant reproduction. Liverworts therefore appear as a critical lineage with respect to terrestrial trait development through the origin and diversification of TF genes regulating specialized functions in reproduction. The evolution of these TF families in *Marchantia* may allow the activation of gene expression during male/female reproductive organ formation and differentiation. However, two TF families present in lower plants and green and red algae did stop with *Marchantia* and were not inherited in higher plants.

This study identifies TF genes and provides a detailed analysis of TF gene expression as a means of understanding the impact of TF diversification on the evolution of liverworts and their importance in the origin of modern land plants from bryophytes to flowering plants. Thus, we have demonstrated the utility of short read sequence data to characterize TF-encoding transcripts using *Marchantia* as a basal lineage in the context of genetic change in a broad comparison of terrestrial plants with their charaphytic and algal ancestors. Further analysis is expected to increase our knowledge of organism diversification through further chromosomal sequence analysis and reorganization. In addition, the identification of *cis*- and *trans*-acting elements associated with plant TFs are expected to reveal additional mechanisms that regulate gene expression in a more tightly regulated genetic context. Future studies are expected to build on the current liverwort TF gene transcriptome through construction of a broader interactome (protein-protein interaction) and elucide the regulons controlling each TF. The establishment of such a TF interactome within a fairly short time span is a feasible and important goal. Such an interactome will encompass TF-TF interactions directly as well as TF-DNA interactions and will highlight the underlying complexity of gene regulation in liverworts.

## Methods

### Plant material and growth conditions

Male and female *M. polymorpha* plants were collected from local wild colonies growing in nurseries in Melbourne, Australia. Male and female lines for RT-PCR and Real-time PCR experimental purposes were established from a single gemma of the thallus. Plants were maintained and propagated in growth cabinet with temperature of 20°C and continuous white light 60 μmol photon m^-2^ s^-1^ and far-red (FR) light 730 nm. Tissues were collected for the RNA sequencing from male and female vegetative thallus (VM and VF), immature male and female reproductive structures (antheridial and archegonial discs) - 2 mm in height (IMM and IMF) and mature male and female reproductive structures (antheridial and archegonial discs) > 2 mm in height (MM and MF) as shown in Additional file 1.

### RNA sequencing and assembly

Total RNA was extracted from the male and female vegetative thalli and immature and mature reproductive gametophytic tissues of *M. polymorpha* (obtained from nurseries across Melbourne) using an RNeasy extraction kit (Qiagen, Australia), according to the manufacturer’s recommendations. RNA samples were quantified using a Nanodrop ND-1000 spectrophotometer (Biolabgroup, Australia). RNA sequencing was performed by the Beijing Genome Institute (BGI), China. In total, six cDNA paired-end libraries were generated using the mRNA-Seq assay for transcriptome sequencing on Illumina HiSeq™ 2000 platform.

Briefly, beads with Oligo(dT) were used to isolate poly(A) mRNA from the total RNA preparations. mRNA was fragmented into short fragments and taking these fragments as templates, random hexamer-primer was used to synthesize the first strand cDNA. The second-strand cDNA was synthesized using dNTPs, RNaseH and DNA polymerase I. Short fragments were purified and resolved for end reparation and adding poly(A). Short fragments were then connected with sequencing adapters and suitable fragment were selected using agarose gel electrophoresis for the PCR amplification as templates. At last, the library could be sequenced using Illumina HiSeq™ 2000.

Raw sequence reads were filtered for low quality reads trimmed off 3’ adaptor sequences. All short read assemblies were performed using publicly available programs: Velvet (version 1.1.05; http://www.ebi.ac.uk/~zerbino/velvet/), developed for de novo short read assembly using de Bruijn graphs [32], and Oases (version 0.1.22; http://www.ebi.ac.uk/~zerbino/oases/), a *de novo* transcriptome assembler for very short reads [33]. After velvet assembly, the resulting contigs were clustered into small groups, loci using Oases to produce transcript isoforms. Various parameters of these programs i.e. K-mer length = 49, N50 length were optimized to obtain the best assembly results with our dataset.

### Similarity search and identification of TF-encoding transcripts

For the identification of TF-encoding transcripts in *M. polymorpha*, all of the assembled transcripts were subjected to a homology search (BLASTX) with known transcription factors (TFs) and other transcriptional regulators (TRs), as classified in Plant Transcription Factor Database (PlnTFDB; version 3.0; http://plntfdb.bio.uni-potsdam.de/v3.0/[40,108]), with an e-value cut-off of 1e-05 using default parameters. PlnTFDB is an integrative database that provides complete sets of TFs and TRs in plant species, which have completely sequenced and annotated genomes and that are listed in the database.

Protein sequences for all of the genes from 20 species listed in the PlnTFDB were downloaded from (http://plntfdb.bio.uni-potsdam.de/v3.0/downloads.php); the file contained 29,473 sequences. This file acted as the database for the local BLASTX search, and the query file contained all the assembled *Marchantia* transcript sequences. The BLASTX results were inspected for their top first hits using in-house python script, and thus, putative transcripts of *M. polymorpha* that coded for TFs were identified.

### Comparison of TF-encoding genes in plants and their algal ancestors

In order to better understand the evolution of TFs, comparative studies of TF gene families was carried out between 21 algal and plant species - 20 species were listed in the Plant Transcription Factor Database (PlnTFDB) and *Marchantia* transcripts. We investigated TF gene evolution based on the phylogenetic positions of plants listed in PlnTFDB and by comparing the number of genes coding for a particular TF family in different plant and algal species taken into consideration. Comparative analysis was performed on the number of TF genes by highlighting similarities and differences in TF gene populations among the organisms taken into consideration. The percentages of identified TF genes compared with the total number of protein-encoding genes in the genome were also analyzed for all species. We took into account the events of emergence, halt, expansion and contraction of particular TF gene families by considering the number of genes/transcripts that encoded for a specific TF in various species.

### Expression patterns of TF-encoding transcripts of *M. Polymorpha*

We mapped all of the reads from six libraries onto the non-redundant set of assembled transcripts to quantify the abundance of the transcripts using Bowtie [109] allowing upto 3 mismatches per read. The calculation of transcript expression in each tissue used the RPKM (number of reads per kilobase per million reads) method [110]. The expression value in terms of the RPKM, which corresponded to each transcript in all six tissues, was determined. TF-encoding transcripts were quantified by the formula:

$$\mathrm{RPKM}=\left(10^{6}*C\right)/\left(\mathrm{NL}/10^{3}\right)$$

where RPKM(A) is the expression of transcript A, C is the number of reads that uniquely aligned to transcript A, N is the total number of reads that are uniquely aligned to all transcripts and L is the number of bases on transcript A. The RPKM method eliminated the influence of different gene lengths and sequencing levels on the calculation of gene expression. Therefore, the calculated gene expression could be directly used to compare the difference in gene expression between samples.

### RT-PCR analysis

For the detection of transcripts that were expressed at specific stages as revealed by the assembly and RPKM methods, RT-PCR was carried out. Reverse transcriptase (Superscript™ One step RT-PCR with Platinum® *Taq*, Invitrogen, Australia) reactions were performed using 20 ng of total RNA, according to the manufacturer’s instructions. The cDNA equivalent of 20 ng total RNA was amplified in 10 μl reactions for 45 min at 50°C. The reaction conditions were as follows: pre-denaturation for 2 min at 94°C, followed by 35 cycles of 94°C for 15 s and annealing/extension at 58°C for 30 s, then 72°C for 1 min, followed by a final extension of 1 cycle at 72°C for 5 min. PCR products were run on a 1% (w/v) agarose gel to confirm the size of the amplification products and to verify the presence of a unique PCR product. Total RNA used in RT-PCR and Real-time PCR analysis experiments were extracted from the clean cultures of *Marchantia*. These RNA preparations were entirely independent from the ones used in RNA sequencing. Two technical replicates were done for each of the nine transcripts. Primers suitable for amplification for each transcript were designed using an online tool from Invitrogen, OligoPerfect™ Designer (http://tools.invitrogen.com/content.cfm?pageid=9716). A list of primers used is given in Additional file 11.

### Real-time RT-PCR analysis

Real time PCR for selected TF encoding transcripts was performed in duplicates using Brilliant III Ultra-fast SYBR QPCR Master mix (Agilent Technologies, Mulgrave, Victoria, Australia) according to manufacturer’s instructions involving 3-step PCR cycle. Quantitative expression differences between samples were estimated using cDNA from male and female vegetative, immature and mature reproductive stages, obtained using the Invitrogen Superscript™III First strand cDNA synthesis kit according to manufacturer’s instructions. After purification and measurement, ~50 ng of cDNA from each stage of the 6 developmental stages was used as template for real-time PCR analysis using Brilliant III Ultra-fast SYBR QPCR Master mix. PCR amplifications were performed on the MX3000P real-time PCR instrument (Agilent Technologies, Mulgrave, Victoria, Australia). Data generated was analysed using MxPro software. All experiments were performed with two technical replicates and the RNA preparations were pooled mixtures of several rounds isolations for each sample, and are entirely independent from the ones used in RNA sequencing, hence the preparations itself contained multiple biological replicates. The quantity of cDNA was calculated by software in nanograms for each sample and is plotted onto a graph for reference transcripts - actin and CDPK genes and the transcript that has uniform constant RPKM values in all six stages (Additional file 9). The starting concentration of each transcript in a sample was expressed relative to the starting concentration of reference transcript. For each examined transcript, the ^C_t_ value between each tested sample and reference gene was calculated and plotted onto a graph. A list of primers used is given in Additional file 11.

### Statistical analysis

Data given in Additional file 2 is divided into 6 ranks according to the group of organisms analysed and fed into Statistical Analysis Software (SAS) version 9.2. To test whether the number of genes encoding for TFs differs significantly among organisms (as grouped in ranks); all data were subjected to analysis of variance using PROC GLM of SAS. The sub-ranks nested within rank (i.e. rank (sub-rank)) was used as error term for significant test of ranks and this referred to individual organisms within a rank. Data was log-transformed prior to analysis to meet the assumptions of homogenous and normally distributed residuals. Pair-wise comparisons between ranks were undertaken with the use of Gabriel’s comparison interval (95% confidence intervals). Further analysis was done using the PROC NESTED (SAS) to determine the variance partitioning pattern among different sources of variation (i.e. rank, sub-rank, genes). The Tukey’s Studentized Range (HSD) Test also grouped 6 ranks in A, B, C and D groups according to the similarity between the number of TF-encoding genes for various organisms.

## Availability of supporting data

The sequence datasets sets supporting the results of this article are available at NCBI in the Short Read Archive (SRA) database under accession number SRP029610.

## Competing interests

The authors declare that they have no competing interests.

## Authors’ contributions

Conceived and designed the study: NS, PB, MS. Performed computational analysis and experiments: NS. Wrote the paper: NS, PB, MS. All authors have read and approved the manuscript.

## Supplementary Material

Additional file 1 **Developmental stages of *Marchantia polymorpha *** **selected for RNA-Seq.** VM (male vegetative thallus), VF (female vegetative thallus), IMM (immature reproductive male), IMF (immature reproductive female), MM (mature reproductive male) and MF (mature reproductive female). Immature male and female reproductive structures (antheridial and archegonial discs) – 2 mm in height and mature male and female reproductive structures (antheridial and archegonial discs) > 2 mm in height are taken into consideration for experimental purposes. Click here for file

Additional file 2 **Number of TF encoding genes in 20 organisms taken into consideration in the study.** Based on the published reports, TF encoding genes in 20 organisms is recorded in the table. Organisms are classified into broader categories: red algae, green algae, liverwort, moss, spike moss, monocots and dicots. Liverwort data is the result of our study. All these classes of organisms are grouped in 6 ranks for statistical analysis. Ranks are also displayed in the table. Bar graph is also plotted for this distribution as shown in Figure 1. Click here for file

Additional file 3 **Transcription factor families and their potential function.** 85 TF families and their functions as listed on Plants Transcription Factor Database (http://plntfdb.bio.uni-potsdam.de/v3.0/). Click here for file

Additional file 4 **24 TF families that evolved with *Marchantia*.** Functions of 24 TF families that arose with emergence of *Marchantia*. TF families highlighted in yellow play role in sexual reproduction. Click here for file

Additional file 5 **RPKM values of *Marchantia *** **transcripts encoding TFs in 6 developmental tissues.** Sheet 1: List of 3,471 transcripts with details of their length and RPKM values in six developmental tissues in *Marchantia*. Sheet 2: List of 96 TF encoding transcript with specifically express in only one tissue. Click here for file

Additional file 6 **List of transcripts expressing specifically in vegetative and reproductive stages.** List of transcripts with details of their length, TF family they are encoding and RPKM values in six developmental tissues in *Marchantia*. Click here for file

Additional file 7 **Table showing nature of correlation coefficients between different gene pairs in each TF family.** List of number of gene pairs with significant positive, negative correlation and no significant correlation between expression patterns of genes in each TF family. Click here for file

Additional file 8 **Top hits of BLASTX of TF encoding transcripts against nr database.** List of transcripts with their top hits when blasted against NCBI nr database. Click here for file

Additional file 9 **Expression profiles of Actin and CDPK genes of *Marchantia *** **and the reference transcript.** In qPCR analysis, the quantity of cDNA was calculated by software MaxPro in nanograms for each sample and is plotted onto a graph for reference transcripts - actin and CDPK and for the transcript that has uniform constant expression in all six stages. Click here for file

Additional file 10 **Statistical results showing analysis of variance.** Organisms under consideration are divided into 6 ranks as red algae – rank 1, green algae – rank 2, liverworts – rank 3, moss and spike moss – rank 4, monocots- rank 5 and dicots – rank 6. All data of TF-encoding transcripts is then fed into SAS to test whether the number of genes encoding for TFs differs significantly among organisms (as grouped in ranks). The Tukey’s Studentized Range (HSD) Test results also show the grouping of ranks 1, 2, 3, 4, 5 and 6 in 4 groups A, B, C and D on the basis of difference in mean. Higher plants (monocots (5) and dicots(6)) are grouped together as A, red algae and green algae are grouped together as D. Liverworts and mosses form the separate groups B and C respectively between the two extreme groups A and D. Click here for file

Additional file 11 **List of primer sequences used for RT-PCR and real-time PCR experiments.** Forward and Reverse primer sequences used in PCR. Click here for file
