# Supplementary material for: Transcriptome-wide profiling and expression analysis of transcription factor families in a liverwort, Marchantia polymorpha
Source: BMC Genomics. 2013 Dec 23;14:915. doi: 10.1186/1471-2164-14-915 (PMC3880041; doi:10.1186/1471-2164-14-915)
Supplement: Additional file 1 — Developmental stages of Marchantia polymorpha selected for RNA-Seq. VM (male vegetative thallus), VF (female vegetative thallus), IMM (immature reproductive male), IMF (immature reproductive female), MM (mature reproductive male) and MF (mature reproductive female). Immature male and female reproductive structures (antheridial and archegonial discs) – 2 mm in height and mature male and female reproductive structures (antheridial and archegonial discs) > 2 mm in height are taken into consideration for experimental purposes. [file 1471-2164-14-915-S1.pptx]

## Slide 1
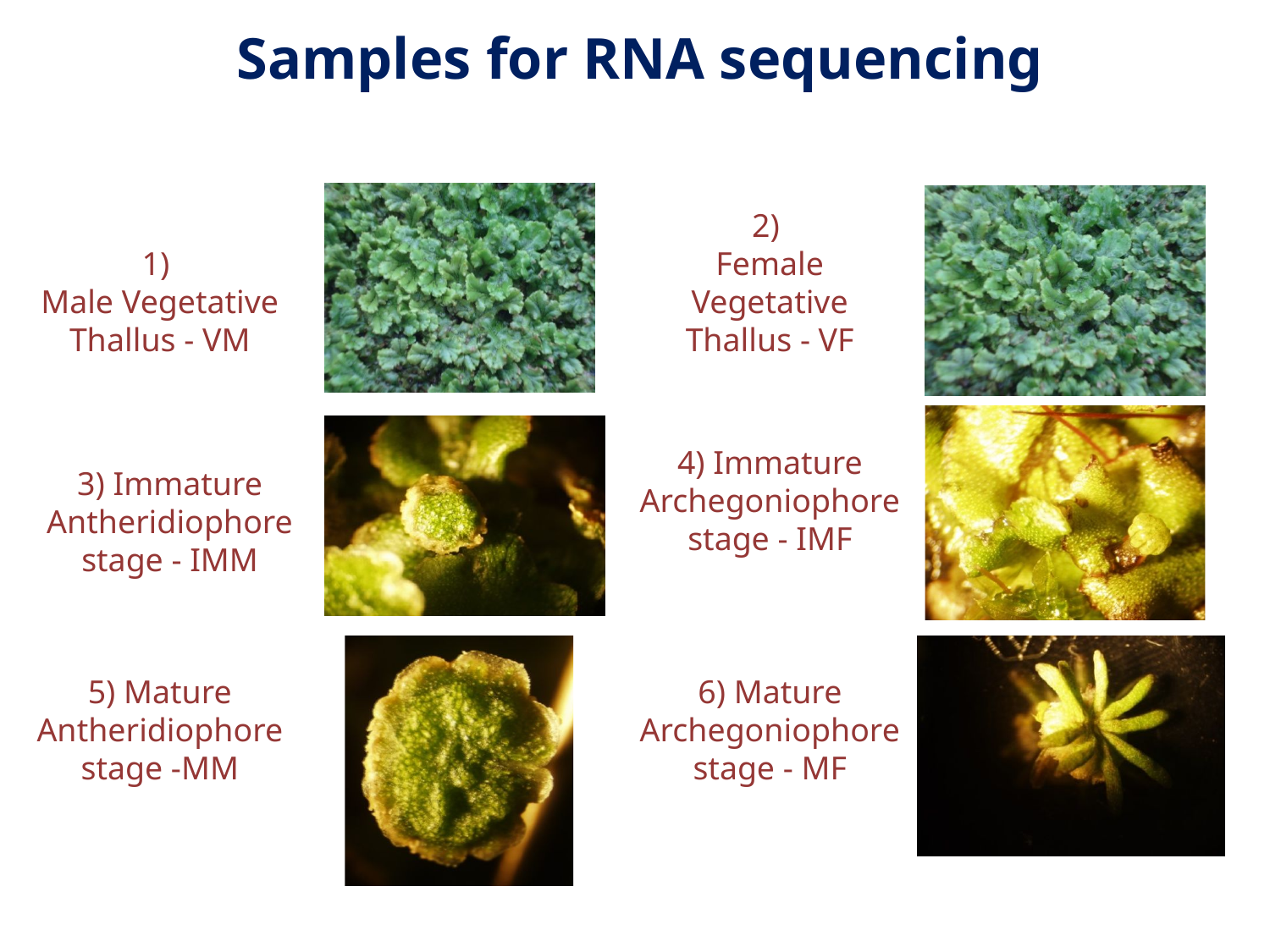

Samples for RNA sequencing
2)
Female Vegetative Thallus - VF
1)
Male Vegetative Thallus - VM
4) Immature Archegoniophore stage - IMF
3) Immature Antheridiophore stage - IMM
5) Mature Antheridiophore stage -MM
6) Mature Archegoniophore stage - MF
