# Supplementary material for: Transcriptome-wide profiling and expression analysis of transcription factor families in a liverwort, Marchantia polymorpha
Source: BMC Genomics. 2013 Dec 23;14:915. doi: 10.1186/1471-2164-14-915 (PMC3880041; doi:10.1186/1471-2164-14-915)
Supplement: Additional file 2 — Number of TF encoding genes in 20 organisms taken into consideration in the study. Based on the published reports, TF encoding genes in 20 organisms is recorded in the table. Organisms are classified into broader categories: red algae, green algae, liverwort, moss, spike moss, monocots and dicots. Liverwort data is the result of our study. All these classes of organisms are grouped in 6 ranks for statistical analysis. Ranks are also displayed in the table. Bar graph is also plotted for this distribution as shown in Figure 1. [file 1471-2164-14-915-S2.pdf]

Additional file 1: Number of TF encoding genes in 20 organisms taken into consideration in the study.

Ranks	1		2							3	4		5				6				
	**Red Algae**		**Green Algae**							**Liverwort**	**Moss**	**Spike moss**	**Monocots**				**Dicots**				
	**Cm**	**Gs**	**Mp**	**Microsomonas**	**Ol**	**Ot**	**Chlorella**	**Cr**	**Coccomyxa**	**Marchantia**	**Pp**	**Sm**	**Osi**	**Osj**	**Sb**	**Zm**	**Al**	**At**	**Cp**	**Pt**	**Vv**
ABI3VP1			1	1			2	1	1	49	30	16	55	65	54	87	73	61	30	83	21
Alfin-like										5	7	1	9	11	11	33	7	9	4	9	6
AP2-EREBP			10	12	9	6	13	14	13	78	149	57	161	196	161	330	149	168	93	210	124
ARF										8	13	7	23	46	25	88	22	35	10	37	19
ARID	3	6	2	4	1	1	3	4	7	9	7	6	6	8	4	16	8	11	7	13	10
ARR-B			1	1	1	1	1	1		4	5	6	8	11	9	18	11	17	10	15	11
AUX/IAA										76	2	4	30	53	26	93	31	36	18	33	23
BBR/BPC										2		1	4	7	5	13	7	16	2	16	2
BES1										12	6	5	7	6	7	18	9	14	5	12	6
bHLH	1	2	2	2	1	1	2	8	4	143	101	46	143	195	148	330	138	177	83	159	102
BSD		2	1	2	2	2	2	2	3	10	9	5	8	11	9	16	8	12	8	12	7
bZIP	3	20	7	10	8	7	8	11	16	49	37	22	89	129	89	238	76	111	41	86	45
C2C2-CO-like				3	2	3	1	1	1	4	11	4	15	23	15	28	17	22	8	16	12
C2C2-Dof			1	1	2	2	1	1	1	12	20	31	28	37	30	57	34	45	18	41	26
C2C2-GATA	6	8	8	6	9	4	7	8	4	17	12	7	30	32	30	64	28	37	21	36	20
C2C2-YABBY													7	15	6	38	6	8	6	13	7
C2H2	7	3	14	16	4	4	4	5	3	153	59	38	102	131	105	204	103	112	69	113	76
C3H	7	16	20	30	24	19	29	22	16	319	45	46	71	109	57	219	75	85	45	99	60
CAMTA										10	1	1	7	7	7	9	6	9	2	7	4
CCAAT	6	8	10	10	9	9	14	8	15	25	27	17	44	71	42	107	52	72	23	59	33
Coactivator p15	1	1	2	1	1	1	1	1	1	6	2	2	2	3	2	7	3	7	3	3	2
CPP	2	3	2	1	2	2	3	2	2	7	6	4	11	20	8	27	7	9	4	12	6
CSD			1	4	3	4	3	2	3	12	3	4	4	3	2	6	17	4	3	7	3
DBP										17		2	6	7	6	14	4	5	1	10	4
DDT	1							1	1	12	3	5	6	8	5	18	4	5	4	5	6
E2F-DP	5	7	3	4	3	3	3	3	3	10	10	4	9	12	11	23	7	14	5	10	6
EIL										10	2	6	8	11	7	13	6	6	3	6	4
FAR1										284			129	138	47	40	5	24	25	42	42
FHA	2	6	11	11	9	7	14	12	6	143	15	9	16	24	15	26	18	19	12	18	14
G2-like	1	1	3	3	2	2	8	4	3	21	41	20	47	56	47	94	44	56	34	66	38
GeBP										1			5	6	7	15	18	20	4	7	3
GNAT	19	20	30	35	22	26	35	33	23	82	44	26	45	42	35	77	37	41	27	54	32
GRAS										17	39	48	57	65	73	112	35	36	38	98	46
GRF										7	2	4	13	19	7	30	9	9	6	9	7
HB	5	2	10	8	6	6	2	1	1	69	43	29	97	140	71	268	80	109	63	130	81
HMG			9	10	7	5	4	7	4	20	9	6	12	17	9	33	12	21	8	12	7
HRT										6	7	2	1	1	1	6	2	2	2	1	1
HSF	3	6	4	6	1	1	1	2	1	4	8	7	26	42	24	57	29	25	18	31	19
IWS1		1	1	1	1	1	1	1	1	1	1	1	1	1	1	7	2	2	1	2	1
Jumonji	3	2	13	10	10	5	11	6	12	23	9	10	14	16	19	40	18	18	14	20	15
LFY										2	2	1	2	1	1	4	1	1	1	1	1
LIM	1	1	1	1	1	1				17	3	3	6	7	5	17	6	10	4	13	5
LOB										46	29	15	43	39	36	88	46	49	34	57	45
LUG										21	1	2	7	12	5	15	2	7	4	5	3
MADS	1	1	1	1	1	1		2	2	114	22	17	73	97	70	142	79	132	139	111	60
MBF1		1		1			1	1	1	3	3	1	4	3	2	12	3	3	2	3	3
MED6	1	1					1			2	1	1	1	1	1	12	1	1	1	1	1
MED7		1	1	1	1	1	1	1	1	1	1	2	1	3	1	6	3	3	1	2	1
mTERF	1		3	2	1	1	2	1		73	13	11	29	37	32	39	42	36	21	51	21
MYB	9	13	7	14	12	10	4	10	8	53	62	22	127	127	122	247	147	166	68	213	130
MYB-related	21	22	16	18	16	16	14	17	14	189	46	45	85	115	85	190	65	100	63	101	68
NAC										107	32	20	145	147	112	216	109	126	70	167	79
NOZZLE										1							1	1	1	2	1
OFP										7	8	6	30	31	36	55	22	18	9	26	11
Orphans	9	12	19	23	10	10	14	19	18	161	118	44	98	102	79	240	91	100	55	148	71
PBF-2-like			1	1	1	1	1	1	1	7		1	2	2	2	8	3	4	2	3	2
PHD	7	5	22	20	14	10	21	23	22	186	47	37	41	63	55	91	47	59	42	70	44
PLATZ	1		2	1	1	1	2	3	1	7	13	14	15	21	16	22	12	14	9	20	9
Pseudo ARR-B			1		2	1	1	1	1	5	2	1	4	14	4	4	5	5	4	7	5
RB	1	1	1	1	1	1	2	1	2	5	2	2	2	4	4	19	1	1	1	1	2
Rcd1-like	1	1	1	1	1	1	1	2	1	2	3	1	6	5	3	12	3	3	2	3	2
RWP-RK	1	9	4	6	3	4	8	12	5	15	8	16	12	14	14	32	16	16	5	18	8
S1Fa-like								1		1	1		3	2		3	3	3	1	2	2
SAP												1					1	1	1	1	1
SBP			2	3			31	23	3	41	14	11	17	29	19	62	15	28	11	29	18
SET	6	6	18	16	16	10	14	14	17	134	26	36	31	36	40	100	35	46	20	44	32
Sigma70-like	4	4	2	1	1	1	1	1	1	8	5	3	7	8	6	15	6	6	3	9	6
SNF2	13	12	22	27	20	20	18	17	27	139	35	32	40	49	38	72	38	45	20	48	33
SOH1		1	1	1	1	1	1	1	1	1	1	1	2	3	2	3	1	1	1	2	
SRS										2	2	4	5	6	6	18	9	13	4	10	4
SWI/SNF-BAF60b	2	2	1	1			2	2	2	7	7	6	10	11	13	39	18	17	9	20	14
SWI/SNF-SWI3	1		1		2	1				10	5	2	4	4	3	5	5	5	6	7	5
TAZ			1	2		1	1	4	1	6	5	2	2	6	4	12	7	8	5	4	3
TCP										7	6	6	22	24	27	65	25	31	21	33	19
Tify										33	12	10	16	23	16	60	16	26	9	17	11
TIG		1	9	10	7	7		2		76											
TRAF	2	3	3	3	2	2	4	32	6	104	25	12	90	79	72	66	31	29	17	30	19
Trihelix										57	25	22	22	26	21	55	24	27	25	43	28
TUB			1	1	1	1	2	2	2	8	6	6	13	26	13	43	11	16	6	11	7
ULT													2	2	1	5	2	2	3	2	1
VARL								12		1											
VOZ											2		2	2	2	9	3	3	2	4	2
WRKY			2	2	1	2	1	1	1	74	37	19	111	129	94	202	79	88	48	104	59
zf-HD										1	8	7	14	15	13	30	17	18	10	23	12
Zn-clus		2																			
