# Supplementary material for: Transcriptome-wide profiling and expression analysis of transcription factor families in a liverwort, Marchantia polymorpha
Source: BMC Genomics. 2013 Dec 23;14:915. doi: 10.1186/1471-2164-14-915 (PMC3880041; doi:10.1186/1471-2164-14-915)
Supplement: Additional file 9 — Expression profiles of Actin and CDPK genes of Marchantia and the reference transcript. In qPCR analysis, the quantity of cDNA was calculated by software MaxPro in nanograms for each sample and is plotted onto a graph for reference transcripts - actin and CDPK and for the transcript that has uniform constant expression in all six stages. [file 1471-2164-14-915-S9.pptx]

## Slide 1
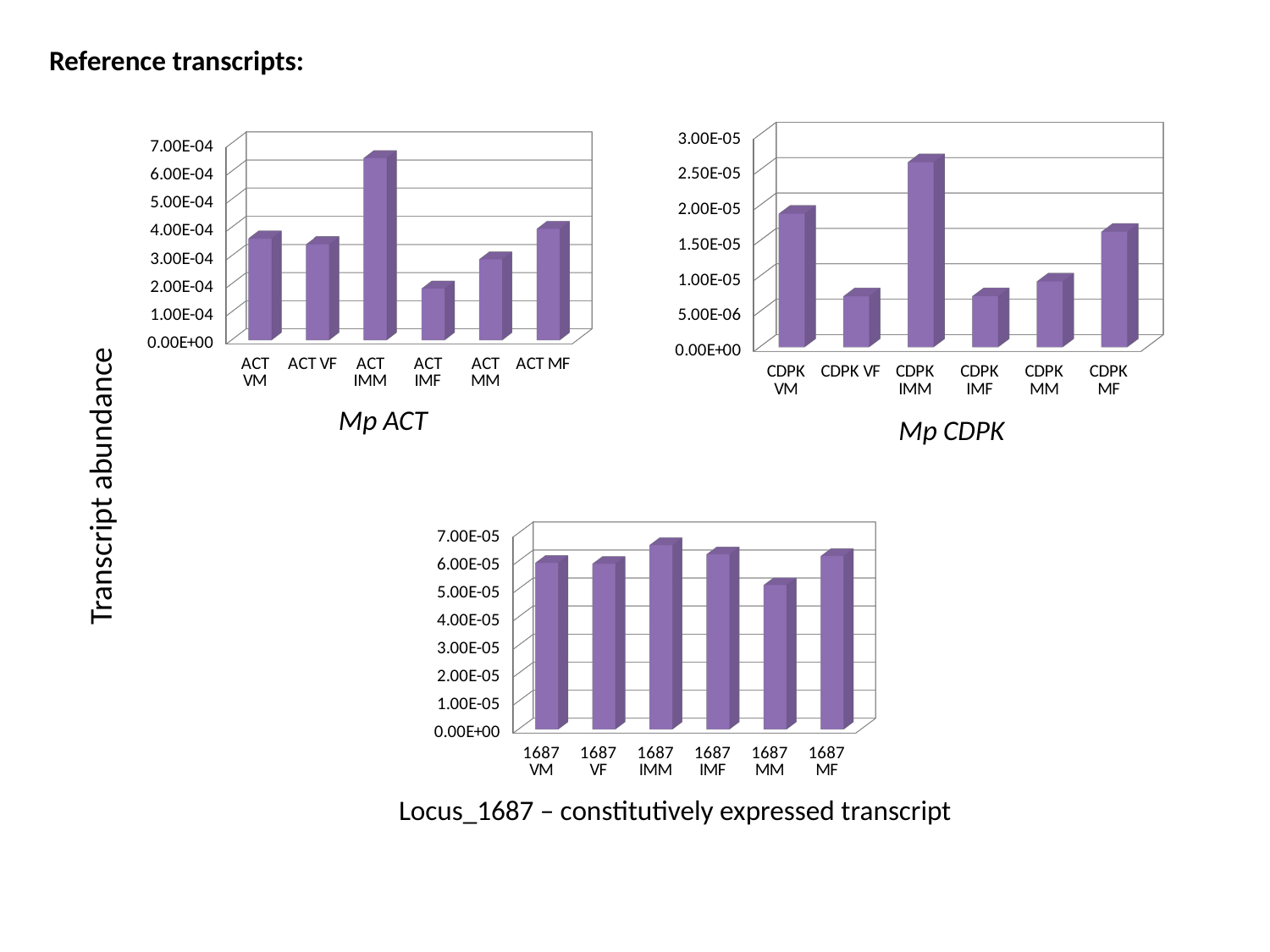

Reference transcripts:
[unsupported chart]
[unsupported chart]
Mp ACT
Mp CDPK
Transcript abundance
[unsupported chart]
 Locus_1687 – constitutively expressed transcript
