# Supplementary material for: Transcriptome-wide profiling and expression analysis of transcription factor families in a liverwort, Marchantia polymorpha
Source: BMC Genomics. 2013 Dec 23;14:915. doi: 10.1186/1471-2164-14-915 (PMC3880041; doi:10.1186/1471-2164-14-915)
Supplement: Additional file 10 — Statistical results showing analysis of variance. Organisms under consideration are divided into 6 ranks as red algae – rank 1, green algae – rank 2, liverworts – rank 3, moss and spike moss – rank 4, monocots- rank 5 and dicots – rank 6. All data of TF-encoding transcripts is then fed into SAS to test whether the number of genes encoding for TFs differs significantly among organisms (as grouped in ranks). The Tukey’s Studentized Range (HSD) Test results also show the grouping of ranks 1, 2, 3, 4, 5 and 6 in 4 groups A, B, C and D on the basis of difference in mean. Higher plants (monocots (5) and dicots(6)) are grouped together as A, red algae and green algae are grouped together as D. Liverworts and mosses form the separate groups B and C respectively between the two extreme groups A and D. [file 1471-2164-14-915-S10.docx]

| **Source** | **DF** | **Type I SS** | **Mean Square** | **F Value** | **Pr > F** |
| --- | --- | --- | --- | --- | --- |
| **Rank** | 5 | 256.9507545 | 51.3901509 | 187.36 | <.0001 |
| **Rank (Subrank)** | 15 | 14.2774170 | 0.9518278 | 3.47 | <.0001 |

| **Tests of Hypotheses Using the Type I MS for Rank (Subrank) as an Error Term** | | | | | |
| --- | --- | --- | --- | --- | --- |
| **Source** | **DF** | **Type I SS** | **Mean Square** | **F Value** | **Pr > F** |
| **Rank** | 5 | 256.9507545 | 51.3901509 | 53.99 | <.0001 |

| **Means with the same letter are not significantly different.** | | | | | | | | |  |  |  |  |  |
| --- | --- | --- | --- | --- | --- | --- | --- | --- | --- | --- | --- | --- | --- |
| **Tukey Grouping** | | | | **Mean** | | **N** | **Rank** | |  |  |  |  |  |
|  | A | | | 1.22230 | | 340 | 5 | |  |  |  |  |  |
|  | A | | |  | |  |  | |  |  |  |  |  |
|  | A | | | 1.10814 | | 425 | 6 | |  |  |  |  |  |
|  |  | | |  | |  |  | |  |  |  |  |  |
| B |  | | | 1.02485 | | 170 | 3 | |  |  |  |  |  |
|  |  | | |  | |  |  | |  |  |  |  |  |
|  | C | | | 0.80890 | | 85 | 4 | |  |  |  |  |  |
|  |  | | |  | |  |  | |  |  |  |  |  |
|  | D | | | 0.38781 | | 595 | 2 | |  |  |  |  |  |
|  | D | | |  | |  |  | |  |  |  |  |  |
|  | D | | | 0.27694 | | 170 | 1 | |  |  |  |  |  |
| **Nested Random Effects Analysis of Variance for Variable Gene** | | | | | | | | | | | | | |
| **Variance Source** | | **DF** | **Sum of Squares** | | **F Value** | | | **Pr > F** | | **Error Term** | **Mean Square** | **Variance Component** | **Percent of Total** |
| **Total** | | 1784 | 755.059365 | |  | | |  | |  | 0.423240 | 0.464434 | 100.0000 |
| **Rank** | | 5 | 256.950755 | |  | | |  | |  | 51.390151 | 0.182182 | 39.2267 |
| **Subrank** | | 15 | 14.277417 | |  | | |  | |  | 0.951828 | 0.007971 | 1.7163 |
| **Difference (among gene numbers)** | | 1764 | 483.831193 | |  | | |  | |  | 0.274281 | 0.274281 | 59.0570 |
